# The Emerging Role of Metabolism in Brain-Heart Axis: New Challenge for the Therapy and Prevention of Alzheimer Disease. May Thioredoxin Interacting Protein (TXNIP) Play a Role?

**DOI:** 10.3390/biom11111652

**Published:** 2021-11-08

**Authors:** Lorena Perrone, Mariarosaria Valente

**Affiliations:** 1 Department of Advanced Medical and Surgical Sciences, University of Campania Luigi Vanvitelli, 80131 Naples, Italy; 2 Department of Medicine, University of Udine, 33100 Udine, Italy; mariarosaria.valente@uniud.it; 3 Clinical Neurology Unit, Department of Neuroscience, Azienda Sanitaria Universitaria Friuli Centrale, University Hospital, 33100 Udine, Italy

**Keywords:** brain-heart axis, metabolism, cardiovascular diseases, TXNIP, microbiota

## Abstract

Alzheimer disease (AD) is the most frequent cause of dementia and up to now there is not an effective therapy to cure AD. In addition, AD onset occurs decades before the diagnosis, affecting the possibility to set up appropriate therapeutic strategies. For this reason, it is necessary to investigate the effects of risk factors, such as cardiovascular diseases, in promoting AD. AD shows not only brain dysfunction, but also alterations in peripheral tissues/organs. Indeed, it exists a reciprocal connection between brain and heart, where cardiovascular alterations participate to AD as well as AD seem to promote cardiovascular dysfunction. In addition, metabolic dysfunction promotes both cardiovascular diseases and AD. In this review, we summarize the pathways involved in the regulation of the brain-heart axis and the effect of metabolism on these pathways. We also present the studies showing the role of the gut microbiota on the brain-heart axis. Herein, we propose recent evidences of the function of Thioredoxin Interacting protein (TXNIP) in mediating the role of metabolism on the brain-heart axis. TXNIP is a key regulator of metabolism at both cellular and body level and it exerts also a pathological function in several cardiovascular diseases as well as in AD.

## 1. Introduction

Alzheimer’s disease (AD) is the most common form of dementia characterized by progressive memory loss and cognitive decline [1]. Its pathophysiology consists in the accumulation of extracellular amyloid (Aβ) plaques and intracellular neurofibrillary tangles, which are composed by hyperphosphorylated Tau [2]. AD includes familiar AD (FAD), due to inheritance of autosomal dominant mutations in genes encoding for proteins involved in Aβ production: amyloid precursor protein (APP) or presenilins (PSEN1 and PSEN2). The discovery of the genes responsible for FAD contributed to the postulation of the amyloid cascade hypothesis of AD pathogenesis, which defines a linear causality between Aβ accumulation and AD pathophysiology [3]. However, FAD constitutes only 5% of total cases of AD, while the large majority are late onset AD (LOAD) [4]. The molecular mechanisms responsible for LOAD are not yet fully elucidated. Several risk factors promote LOAD, with age as the major risk factor [4]. Up to now, only four FDA approved drugs are currently employed in AD patients: memantine, a N-methyl-D-aspartate (NMDA) blocker, and three inhibitors of the cholinesterase (ChEIs): donepezil, galantamine, and rivastigmine. Notably, each drug alone or a combination of ChEIs with memantine, only modestly ameliorate the cognitive deficit in AD patients. It has been developed only another drug for AD, Aduhelm (aducanumab), very recently approved by FDA [5]. This drug is an Aβ-directed antibody and counteracts Aβ accumulation and its downstream effect. However, the efficacy of such treatment is still debated [6]. A critical point for the discovery of new therapeutic strategies consists in the still incomplete understanding of the specific causes of AD. Thus, it is necessary discover new therapeutic targets for AD. For this reason, several studies are aimed in unveiling the role of metabolism and risk factors in AD onset and progression. In particular, cardiovascular diseases (CVDs) are associated to cognitive decline and participate to AD [7]. In addition, the risk factors for CVDs promote also AD: hypertension [8], diabetes [9], obesity [10], dyslipidemia [11]. In agreement, Asymmentric DiMethyl Arginine (ADMA) plasma levels, which is an excellent biomarker for sporadic small vessels disease (SVD) and indicates early endothelial dysfunction [12], is also a blood biomarker for AD [13] and its levels correlate with cognitive decline [14].

Several studies reveal that AD is a systemic disease. APP is expressed not only in brain but also in peripheral tissues and Aβ produced in peripheral tissues participates to AD [15]. In agreement, the Receptor for Advanced Glycation Endproducts (RAGE), which transports peripheral Aβ into the brain across the Blood Brain Barrier (BBB), plays a key role in AD [4]. In addition, Aβ accumulates also in peripheral tissues, contributing to AD pathogenesis [16]. Notably, Aβ accumulation in cardiomyocytes parallels cardiac diastolic dysfunction in early onset AD, while in late-onset AD it is associated to thickening of the left ventricle wall, suggesting a bidirectional link between AD and cardiac dysfunction [17]. In agreement, recent studies underline a connection between neurodegenerative and cardiovascular diseases, defining the so-called brain-heart axis [18].

In this review we describe the molecular pathways implicated in brain-heart axis and their role in AD. Moreover, we summarize the role of metabolism in altering the brain-heart axis, participating to AD. We summarize also the bi-directional regulation between the composition of the gut microbiota and the pathways modulating the heart-brain axis, impacting on AD progression. We underline the role of these data for the discovery of new molecular targets in AD and the set-up of new therapeutic strategies. We shed light into the potential role of Thioredoxin Interacting Protein (TXNIP)—the inhibitor of the ROS-scavenger Thioredoxin (Trx) and the regulator of glucose homeostasis [19]—as key regulator of the brain-heart axis and thereby a therapeutic target for AD, by analyzing the putative role of TXNIP in the bi-directional regulation of the brain-heart axis in AD.

## 2. TXNIP: Its Function on Metabolism and Brain-Heart Axis

TXNIP is also called Thioredoxin Binding protein 2 (TBP-2) and Vitamin D Upregulated Protein 1 (VDUP-1) [20]. TXNIP is a 46-kDa ubiquitous protein constituted by 391 amino acid residues and is encoded on human chromosome 1q21.1. TXNIP is the endogenous inhibitor of Trx and induces oxidative stress [20]. Hyperglycemia in vivo and high glucose conditions (HG) in vitro promote TXNIP expression [21], TXNIP plays a pathological role in both type 1 and Type 2 diabetes [20] and is also involved in the pathophysiology of diabetic complications, including diabetic retinopathy [22]. TXNIP possesses an α-arrestin domain, which promotes the intracellular trafficking of plasma membrane associated proteins and drives the transport of proteins in different subcellular localizations [23]. Moreover, the α-arrestin domain is responsible for the Trx-independent functions of TXNIP. TXNIP is a shuttle protein that can translocate in different subcellular compartments [23]. In the majority of the cells in normal conditions, TXNIP shows a nuclear localization. However, following different stimulations, TXNIP can translocate in other subcellular compartments, where it exerts specific functions [23]. TXNIP may translocate into the mitochondria, where it blocks the ROS scavenger activity of Trx2, leading to oxidative stress [23]. TXNIP also promotes the nuclear translocation of the transcription factor NF-kB, leading to the expression of pro-inflammatory genes [21]. For this reason, TXNIP is considered the link between oxidative stress and inflammation [24]. In the cytoplasm, TXNIP is implicated in the activation of the NLRP3 inflammasome [24]. TXNIP is also a downstream effector of the Receptor for Advanced Glycation Endproducts (RAGE) [25], which mediates the cellular response to pro-inflammatory molecules such as S100 calcium binding proteins family, HMGB1, Advanced Glycation Endproducts (AGEs), and Aβ [4]. Furthermore, TXNIP protein levels are highly regulated by the ubiquitin-proteasome system [23].

TXNIP participates to the progression of several diseases of the central nervous system, such as AD, Parkinson Disease (PD), and stroke [26]. TXNIP is over expressed in the hippocampus of various AD mice models, such as APP/PS1 mice [27], 3Tg AD mice [28], and 5xFAD mice [29]. Microarray experiments demonstrate that TXNIP is one of the genes more over-expressed in the hippocampus of AD patients [28], as well as immuno-histological studies show that TXNIP is over-expressed in the post-mortem hippocampus of AD patients [29]. It has been suggested that TXNIP is implicated in AD by inducing inflammation through the activation of the NLRP3 inflammasome [26]. In agreement, inflammation through RAGE axis plays a key role in AD [30]. Moreover, studies in vitro and in vivo reveal that TXNIP promotes oxidative damage in an AD contest, leading to tau hyperphosphorylations and subsequent alterations on the neuronal cytoskeleton [29]. Our preliminary data show that silencing of TXNIP in an AD mice model prevents inflammation and altered microglia activation. In addition, our preliminary data suggest that TXNIP plasma levels correlates with cognitive decline [31].

Below we discuss the role of TXNIP in modulating various pathways implicated in the brain-heart axis. We describe the molecular pathways triggered by alterations in TXNIP expression/function, which in turn affect the body and cellular metabolism and alter the brain-heart axis, promoting AD.

## 3. The Heart-Brain Axis and the Bi-Directional Connection with AD Role of Txnip

**Figure 1:**
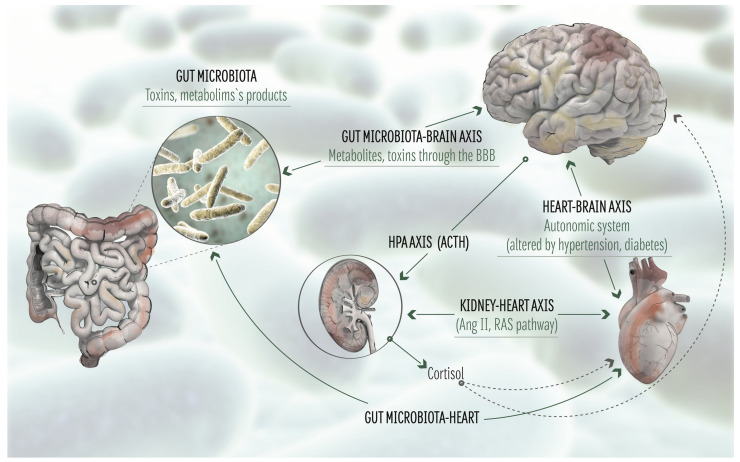
**The microbiota-gut-brain-heart axis.** Schematic representation of the major pathways implicated in the microbiota-gut-brain-heart axis. BBB: Blood Brain Barrier. HPA: Hypothalamic-pituitary-adrenal axis. ACTH: adrenocorticotrophic hormone. Ang II: angiotensin II. RAS: Renin-Angiotensin system.

Several studies demonstrate a connection between AD and cardiovascular diseases. Cardiovascular diseases are risk factors for AD and induce Aβ deposition as consequence of diminished cerebral blood flow and enhanced oxidative stress [16]. In addition, it has been demonstrated a positive correlation between heart failure and cognitive decline [16]. Conversely, genetic mutations within PSEN1 and PSEN2 genes, which are responsible for FAD, give raise also to dilated cardiomyopathy, reinforcing the hypothesis that these diseases share common mechanisms [32]. Notably, ApoE4, the major genetic risk factor for AD, is a risk factor for coronary heart disease [33]. ApoE4 carriers show higher plasma cholesterol concentration, which is a risk factor for both AD and cardiovascular diseases [33]. Interestingly, epigenetic modifications of TXNIP correlate with enhanced risks of cardiovascular diseases [34]. Additionally, the levels of circulating mRNA encoding TXNIP are linked to coronary and heart diseases [35]. Notably, TXNIP is considered a marker of cardiovascular diseases, including myocardial ischemia, hind limb ischemia, and also cerebral ischemia [23]. In addition, TXNIP plays a pathological role in several diseases of the central nervous system, such as AD, Parkinson Disease (PD), and stroke [26]. TXNIP is over expressed in the hippocampus of various AD mice models, such as APP/PS1 mice [27], 3Tg AD mice [28], and 5xFAD mice [29]. Microarray analysis show that TXNIP is one of the genes more over-expressed in the hippocampus of AD patients [28], as well as immuno-histological studies show that TXNIP is over-expressed in the post-mortem hippocampus of AD patients [29]. Below, we summarize the molecular pathways involved AD-related brain-heart axis (Figure 1) and the effect of metabolic AD risk factor on these pathways. We provide evidences that unveil the central role of TXNIP in mediating the effects of metabolism in altering the brain-heart axis and in turn promoting AD. These data, are suggesting a key role of TXNIP in brain-heart axis.

## 4. The Central Autonomic Network (CAN): Effect on AD

The anterior cingulate cortex, parabrachial nucleus, hypothalamus, amygdala, periaqueductal grey matter, the anterior insula and some areas of the medulla regulate the cardiac function. The sympathetic and parasympathetic nervous system mediate the function of these cerebral structures in modulating the cardiac activity (the force of contraction and heart rate), representing the responses to emotional events and stress, as well as the homeostatic reflexes [36]. The autonomic systems (sympathetic and parasympathetic structures, suprachiasmatic nucleus, higher nervous centers including some of those involved in depressive and aggressive behavior) have a key function in the control of heart rate variability (HRV), which is implicated in atherosclerosis, arrhythmias, heart failure, myocardial infarction, and sudden cardiac death [37].

Increased sympathetic activity augments the levels of catecholamines, serotonin, renin, cortisol, aldosterone, angiotensin, and free radicals, promoting dysfunction [37]. On the other hands, elevated parasympathetic activity increases the levels of acetylcholine, dopamine, nitric oxide, endorphins, Q10 coenzyme, which exert a protective function [37]. Elevated levels of acetylcholine are protective for the suprachiasmatic nucleus, modulating the sympathetic activity and in turn lowering the risk of myocardial infarction [37]. Cognitive and autonomic processes are linked through the central autonomic network (CAN), which modulates both the cognitive function and the autonomic regulation of cardiovascular function [38]. The CAN is constituted by a network of cortical and subcortical region, including the insula, hippocampus and prefrontal cortex, which projects to the preganglionic neurons of the autonomic nervous system (ANS). Thus, the CAN is widely considered as the neuroanatomic substrate of a brain–heart axis [39]. The analysis of HRV is extensively used in clinical research for the assessment of autonomic function [40]. It has been demonstrated a correlation between HRV and cognitive function in large cohorts of elderly patients [41], as well as in smaller cohorts of AD patients [42]. Neuroimaging studies show the presence of atrophy and disrupted functional connectivity of hippocampus and insula in subjects with amnestic mild cognitive impairment (aMCI) and AD [43]. Moreover, these alterations correlate with memory dysfunction [44]. The insula plays a key role within the CAN. In animal models, electrical and chemical stimulation of the insula enhance the heart rate (HR) and blood pressure [45]. In humans, insular activity negatively correlated with parasympathetic HRV [46]. Studies are suggesting a connection also from the hippocampus and the sympathetic system. Functional neuroimaging studies in humans reveal the activation of the hippocampus during sympathetic challenges [47]. In addition, parasympathetic HRV negatively correlates respect to hippocampal activity [46]. Recently it has been described a negative correlation between the activity in the hippocampus and a HRV complexity index [48], further supporting the role of hippocampus in the bi-directional effect between AD and CAN, affecting the brain-heart axis.

## 5. Metabolic AD Risk Factors and Central Autonomic Network: A Bi-Directional Regulation

The nervous system is implicated in the pathogenesis of obesity and insulin resistance. In particular sympathovagal imbalance, and the relative prevalence of sympathetic activity seem to play a pivotal role in this bi-directional relationship [49]. Several mechanisms have been reported as link between CAN and obesity and insulin resistance, with a special focus on the role of leptin in modulating the energy expenditure and the sympathetic activity [50].

The autonomic network modulates the body weight both in the short and long term through the vagal nervous afferents, which connect the gut to the brain, regulating the sense of satiety and the food intake [51]. Insulin resistance and sympathetic activity also show a bi-directional regulation. Insulin resistance is associated to enhanced basal sympathetic activity that correlates with the degree of insulin resistance, leading to hypertension [52]. Insulin activates the sympathetic activity directly in the brain. During fasting, low plasma levels of insulin lowers insulin-mediated glucose metabolism in hypothalamic neurons, promoting an inhibitory pathway that blocks chronically active sympathetic centers in the brain stem. After carbohydrate intake, the augmented insulin plasma levels promote insulin-mediated glucose metabolism in the same neurons, enhance the glucose metabolism and reduce the inhibitory pathway, resulting in a stimulatory effect of the sympathetic centers at the brain-stem levels [49]. This regulation promotes hypertension in obese subjects, which show insulin resistance on peripheral glucose uptake but are not resistant to the effect of insulin on the sympathetic system [53]. Thus, this circuit promotes hypertension in the presence of insulin resistance, augmenting the of AD. Interestingly, genetic polymorphisms altering TXNIP expression are associated to hypertension [54], arterial stiffness [55], and enhanced risk to developing coronary heart disease [56]. Notably, TXNIP modulates the glucose homeostasis in a Trx-independent manner [57]. Metabolic signals that promote TXNIP, such as HG, inhibit the glucose uptake through the insulin-responsive glucose transporters. TXNIP induces the endocytosis and degradation of the Glut1 [58] and Glut4 glucose transporters [59], blocking the glucose uptake. TXNIP also regulates the glucose utilization, the mitochondrial oxidation of metabolic substrates, and the gluconeogenesis in the liver [23]. These data are suggesting that TXNIP mediates the effect of hypertension and altered insulin signaling on the brain-heart axis, thereby promoting AD.

Leptin—the product of the obese (ob) gene—is a hormone released by the white adipose tissue and it increases the energy expenditure by stimulating the cardiovascular system and the thermogenesis mainly through the arcuate nucleus (ARC) of the hypothalamus [60]. Leptin plasma levels are reduced during fasting, while augment after overfeeding and regulate energy balance by decreasing appetite and increasing energy expenditure through sympathetic stimulation [61], leading to increased arterial blood pressure. Obesity causes elevated levels of circulating leptin as consequence of leptin resistance. The enhanced leptin plasma levels may augment blood pressure, promoting hypertension [62], which is an additional risk factor for AD. Interestingly, a cross-talk between leptin and insulin response has been demonstrated. Leptin improves glucose homeostasis and insulin response [63]. Thus, leptin resistance in obesity may induce insulin resistance, resulting in altered glucose metabolism and sympathetic activity. In addition, obesity-induced elevated leptin levels promote a significant alteration of the gliovascular interface in the hypothalamus, causing arterial hypertension [64]. Moreover, obesity promotes the hypothalamic expression of the protease Bace1, which produces elevate levels of hypothalamic Aβ peptide and promotes leptin resistance in the hypothalamus [65].

Leptin receptors are encoded by the diabetes (db) gene and by the leptin receptor gene, which can produce six different isoforms: ObRa-f [66]. Leptin is known to play a key function in the hypothalamus. However, the ObR receptors are expressed also in various regions of the hippocampus, in particular at the level of the synapses, where they enhance the synaptic activity following interaction with leptin [67]. Leptin deficient obese mice show a significant impairment of the hippocampal synaptic plasticity, leading to a spatial memory impairment [68]. Leptin exerts a neuroprotective function. Leptin ablation in obese ob/ob mice results in a significant reduction of brain weight, which is ameliorated by leptin treatment [69]. Interestingly, diet is a risk factor for AD [70], suggesting a correlation between diet, metabolic dysfunction and AD. Furthermore, obesity and leptin resistance are risk factors for AD. Leptin resistance leads to elevated leptin plasma levels. However, AD patients show weight loss and concomitant low leptin plasma level [71]. In addition, low circulating leptin is considered a risk for AD [72]. This contradiction results from the effect of leptin on the function of the hypothalamus. Thus, the ability of leptin to regulate food intake and body weight occurs within a tightly regulated concentration range, such that too low circulating leptin fails to maintain energy homeostasis, whereas elevated leptin levels present in the obese state produces leptin resistance and also leptin’s incapacity in regulating food intake [73]. On the other hand, AD is characterized also by not cognitive alterations, such as late-life body weight loss and low body mass index (BMI) [74]. Interestingly, in AD patients as well as in elderly not affected by AD, weight loss and low BMI strongly correlate with AD pathological characteristics: increased amyloid burden [75] and CSF biomarkers of AD [76]. In addition, weight loss is predictive for the transition from amnestic mild cognitive impairment (aMCI) to AD [77]. These data strongly suggest a common mechanism between body metabolism alterations and AD. In agreement, metabolic dysfunction, changes in bodyweight ad altered feeding behavior are present not only in AD patients but also in the 3Tg AD mice model [78]. Moreover, the 5xFAD mice show gliosis associated to hypothalamus dysfunction, altered insulin and leptin signaling in the hypothalamus, which in turn affect the food intake, the energy expenditure, leading to diminished body weight [79].

It is relevant to note that metabolic alterations and weight loss in AD patients occur at least a decade before of the appearance of the first signs of cognitive impairment [80]. For this reason, the understanding of the molecular pathways promoting metabolic dysfunction in AD may provide the discovery of biomarkers essential for the early diagnosis of high risk to develop AD.

The observed metabolic dysfunction in AD may be due to alterations in hypothalamic activity. The hypothalamus is central for the modulation of the energy homeostasis and the feeding behavior. The hypothalamus coordinates the energy homeostasis by balancing the energy expenditure with the food intake. Such function is modulated also by peripheral molecules, such as insulin and leptin, but also of nutrients and their metabolites (e.g., glucose). Notably, the same pathways have a role in AD. Indeed, food intake -via leptin-mediated hypothalamus activity- has a function in AD [81].

Notably, hypothalamic dysfunction occurs early in AD. Pathological features of AD, such as Aβ and tau, are found also in the hypothalamus [82]. Conversely, altered hypothalamic function due to AD is responsible of metabolic dysfunction. In agreement, AD seems to increase the risk to develop type 2 diabetes [83]. Moreover, Aβ oligomers produced in AD alters the function of hypothalamic neurons, leading to peripheral metabolic dysfunction [84]. In turn, AD-induced metabolic dysfunction exerts an impact on the heart function. Thus, there is a bidirectional role of the metabolism in modulating the brain-heart axis in AD onset/progression and the hypothalamus plays a key role in this bi-directional regulation.

Notably, TXNIP plays also a major role in modulating the response to nutrients and in the whole-body energy homeostasis. TXNIP is expressed in the nutrient-sensing neurons of the hypothalamus, where it responds to nutrients and hormonal signals and in turn regulates the adipose tissue metabolism [85]. TXNIP expressed in Agrp hypothalamic neurons regulates the leptin sensitivity of the central nervous system [86]. Considering the role of leptin in modulating the effect of metabolism on the brain-heart axis, we may speculate that TXNIP mediates the effect of metabolism in the brain-heart axis and may act early, before AD onset.

## 6. The Renin-Angiotensin System: Role in AD and AD Risk Factors

The renin-angiotensin system is essential for the regulation of vasoconstriction, blood pressure and cardiovascular homeostasis. Renin converts the angiotensinogen (AGT) into angiotensin I (ANGI), which in turn is converted to angiotensin II (ANGII) by the angiotensin converting enzyme 1 (ACE1). ANGII interacts with the angiotensin receptors (ATRs), modulating various signaling pathways that augment the blood pressure. Renin also induces the conversion of AGT to ANGII through activation of the pro-renin receptor (PRR), which enhances the activity of pro-renin. Short term activation of the renin-angiotensin system is beneficial. On the contrary, its prolonged activation affects the cardiovascular and renal systems, leading to fibrosis and hypertrophy [87]. Notably, there is a brain specific renin-angiotensin system, whose components are expressed and produced in the brain: glial cells, and neurons, with an enhanced expression in the brain areas that are implicated in the modulation of the heart and fluid homeostasis [88]. Brain produces a particular isoform of renin: renin-b [89]. Research data are suggesting that the brain renin-angiotensin system modulates the systemic blood pressure. The knockout of AGT, PRR, or ATRs specifically into the brain blocks hypertension, while overexpression of genes enhancing ANGII production in the brain leads to hypertension in rodents [87]. ANG II produced in the brain by the brain renin exerts various neuronal effects, including the regulation of the sympathetic system and in turn may cooperate in causing the so-called neurogenic hypertension [90]. Thus, it plays a key role in the heart-brain axis. Moreover, patients affected by cardiovascular disease show enhanced activity of brain ACE and ACE inhibitors ameliorate cognitive decline in the elderly [88]. Noteworthy, TXNIP is implicated in ANGII-promoted cardiac fibrosis and hypertrophy and knockdown of TXNIP suppressed ANGII-induced cardiac remodeling [91]. These data further support the role of TXNIP in modulating the brain-heart axis.

Hypertension and diabetes are risk factors for cardiovascular disease and frequently occur together in patients [92]. Diabetes and hypertension are AD risk factor [4]. Interestingly, in diabetes several ANGII downstream effectors are altered, resulting in oxidative stress and inflammation. Notably, chronic ANGII activation produces oxidative stress, which in turn promotes insulin resistance and dyslipidemia, participating to diabetes. In addition, chronic ANGII activity may enhance sodium retention, contributing to hypertension [92]. In AD, the brain renin-angiotensin system causes activation of the microglia, leading to chronic inflammation, which in turn affects the neuronal function [93]. There is a bi-directional regulation between AD and the brain renin-angiotensin system. ANGII enhances the activity of the γ-secretase, leading to an enhanced production of Aβ [94]. Conversely, elevated Aβ production promotes the oligomerization of AT_2_R, which correlates with neurodegeneration [95].

Inhibitors of the renin-angiotensin system are proposed and studied as therapeutic strategy for AD [96]. However, the aim of the present review is the analysis of metabolic factors in the brain-heart axis and their role in AD. As described above, diabetes and hypertension show a bi-directional regulation with the renin-angiotensin system, affecting the heart-brain axis and in turn contributing to AD.

## 7. The Natriuretic Peptides and Endothelins Counteract the Renin-Angiotensin Pathway: A Role in AD?

The natriuretic peptides (NPs) are a family of hormones implicated in the homeostasis of fluid volume and blood pressure. They promote the excretion of sodium, counteracting the renin-angiotensin pathway [97]. NPs consist in 3 groups: atrial natriuretic peptide (ANP), brain natriuretic peptide (BNP), and C-type natriuretic peptide (CNP). NPs interact with three different receptors: (i) Natriuretic Receptor A (NPRA), which binds ANP and BNP; (ii) NPRB that interacts with CNP, and (iii) NPRC, which binds the three NPs groups [97]. NPs are expressed in cardiomyocytes during early development and their expression is activated also in the adult during cardiac remodeling due to stressors [97]. Both NPRA and NPRB are expressed in the brain, but NPRB is the most abundant NPs receptor in the brain [97]. All the NPs proteins are present in the brain. However, the mRNA encoding BNP is absent into the brain, showing that it is expressed in other tissues and then transported into the brain [97]. NPs have neuroprotective effects. Increased brain ANP reduces blood pressure [97]. On the contrary, intracerebroventricular delivery of CNP in sheep depresses blood pressure [97]. It has been shown a significant correlation between brain atrophy and brain BNP levels [98]. Since BNP is not expressed in the brain, these data are suggesting that cardiomyocytes produce BNP in response to brain injury. Interestingly, NPRA is more abundant in the brain of AD patients, while NPRB levels are reduced in the CSF of AD patients [99]. Notably, BNP and N-terminal pro-brain natriuretic peptide (NT-proBNP) are the most important humoral indicators of cardiac function and heart failure [100]. Recent studies are suggesting that natriuretic peptides play a role also in the regulation of the energy metabolism, creating a link between heart and insulin sensitive tissues [101]. It has been suggested that dysfunction of the natriuretic system participates to the development of type 2 diabetes and obesity [101], which are risk factors for AD.

Endothelins is a family of vasoactive peptides, including ET-1, ET-2 and ET-3, which play a relevant function in both cardiovascular functions and dysfunction [102]. ET-1 activates two different G-coupled receptors: ET_A_ and ET_B,_ with ET_A_ mostly localized on vascular smooth muscle cells and pericytes and producing a contractile and proliferative effect following interaction with ET-1 [102]. On the contrary, ET_B_ expressed on endothelial cells leads to vasodilation. Only in pathological conditions ETB is expressed also on vascular smooth muscle cells promoting vasoconstriction [102]. Notably, ET-1 inhibits renin secretion from renal juxtaglomerular cells, modulating the renin-angiotensin system [103]. ET-1 induces cardiomyocytes hypertrophy by activating Protein Kinase C (PKC) [104]. ET-1 promotes cerebrovascular dysfunction by affecting the BBB, leading to BBB breakdown and subsequent inflammation [102]. Several studies indicate that ET-1 is implicated in cognitive impairment and AD by inducing vascular dysfunction [102]. ET-1 levels are increased in AD, leading to vascular dysfunction and inflammation. For this reason, ET-1 is considered a therapeutic target for AD [105]. In agreement, ET-1 interaction with ET_A_ reduces the density and diameters of hippocampal capillaries in APP/PS1 mice, enhancing Aβ deposition. Inhibitions of ET_A_ with ferulic acid ameliorates microvascular dysfunction and Aβ deposition [106]. Finally, it has been proposed a genetic linking between ET-1 and AD prevalence in certain populations that show enhanced ET-1 system, which may predispose to AD [107].

## 8. Role of the Gut Microbiota in the Brain-Heart Axis: Effect of Metabolism

All the microbes present within an ecological niche such as the gut constitute the microbiota, whereas the combination of a defined microbiota and their genes forms the microbiome [108]. The gut microbiota defines a complex ecosystem that includes microbes of all kingdoms (bacteria, fungi, archaea, protozoa, and the meiofauna). Notably, the microbiota establishes an intimate symbiotic relationship with the host [109].

The gut microbiome (GM) participates to the gut-brain axis both directly and indirectly. Several pathways constitute the gut–brain axis. Neural networks regulate the enteric nervous system independently or via the central nervous system (CNS), using the sympathetic efferent in prevertebral ganglia. Another pathway consists in parasympathetic efferent of the vagus nerve [110]. Through these pathways, the GM modulates the hypothalamic–pituitary–adrenal (HPA) axis and the brain function through microbial molecules and metabolites, which act as secondary messengers [111]. The enteric nervous system, which belongs to the autonomic nervous system, modulates the gastrointestinal function and cooperates with the vagal afferent nerves, which in turn transduce sensory information from the gut to the CNS [112]. Several studies underlined the bidirectional regulation in the gut-brain axis.

Dysbiosis defines the alteration of the GM and is characterized by modifications of the composition of bacterial Phyla present in the gut [113]. Several recent studies underline its role in the development of metabolic diseases [114]. GM has an impact on the host health through two pathways: (i) the release of bacterial components, in particular pathogen-associated molecular patterns (PAMPs), which include lipopolysaccharides (LPS) as the most known component [115]: (ii) the production of metabolites derived from the processing of food in the gut. Notably, GM is essential in modulating the permeability of intestinal mucosa [116]. GM exerts a relevant impact on the host’s metabolism. Dysbiosis is associated to altered metabolism and to diseases induced by metabolic dysregulation [117]. Dietary habits play a key role in promoting dysbiosis. The high-fat diet (HFD), which promotes the progression of metabolic syndrome (MS), obesity and type 2 diabetes, causes also dysbiosis. Such condition promotes an augmented permeability of the gut by the GM, leading to an increased LPS serum level, which stimulates inflammation and expansion of the adipose tissue [118], promoting the onset and progression of MS and type 2 diabetes, which are risk factors of AD and of cardiovascular diseases. Moreover, dysbiosis strongly affects the integrity of the BBB, leading to neuroinflammation [119]. As we described before, neuroinflammation affects the CAN. Western diet induces inflammation of the hypothalamus, which in turn promotes leptin resistance and weight gain [120]. Notably, depletion of the GM in mice fed with HFD results in a diminished inflammation of the hypothalamus and improves leptin sensitivity [120]. Interestingly, GM depleted mice fed with HFD show enhanced levels of glucagon-like peptide 1 (GLP-1), which is essential for GM depletion-induced amelioration of hypothalamus inflammation [120] through the signaling downstream GLP-1 receptor (GLP-1R) present on astrocytes [120]. These data further demonstrate the role of GM in modulating the function of the hypothalamus and the downstream regulatory cascade.

In particular, it has been described a bidirectional regulation between the GM and the CAN [110]. The gut-brain axis is mediated by the vagus nerve [110]. The afferent fibers of the vagus nerve act as a sensor for the metabolites released by the GM and transduce the alterations of the GM metabolites to the brain. Conversely, the vagus nerve can modify the GM composition by the cholinergic efferents, which release an anti-inflammatory signal. In agreement, diminished vagal tone leads to dysbiosis [110]. Furthermore, enhanced sympathetic activity is correlated to dysbiosis, increased gut permeability and inflammation [121]. Moreover, GM and sympathetic alterations promote high blood pressure in an animal model of hypertension [122]. In this animal model, reduction of the sympathetic activity, using acethylcoline esterase inhibitors, reduces the blood pressure, ameliorates the dysbiosis and gut permeability [122]. In addition, dysbiosis leads to autonomic imbalance and activates the sympathetic system, promoting inflammation [123]. Interestingly, dysbiosis affects also the renin-angiotensin system, promoting vascular dysfunction through angiotensin II [124]. Thus, dysbiosis affects also the cardiovascular system via a GM-dependent gut-brain-heart axis. Notably, activation of the angiotensin converting enzyme 2 can diminish the immune response by altering the GM, suggesting that alterations of the renin-angiotensin system due to MS and type 2 diabetes promote inflammation by altering the GM [125].

During the course of aging, the gastrointestinal tract epithelial barrier and the BBB become significantly more permeable [126]. Thus, the CNS becomes more susceptible to potential neurotoxins generated by the GM. Microbial insults contribute to AD, by promoting a pathological cascade throughout the vagus nerves to the CNS. Moreover, GM can be affected by other pathological agents, such as fungal or viral infections. Such altered GM may contribute to AD [111]. AD patients show chronic fungal infections and disseminated diffuse mycoses, further confirming that altered microbiome contribute to AD [127]. Altered GM can prime the immune system, which in turn become activated by the brain Aβ [128]. Exposure to Manganese (Mn) can promote AD progression by inducing neuroinflammation. Notably, the transplantation of fecal microbiome derived from control rats to rats exposed to Mn reduces neuroinflammation and Aβ production, confirming the role of MG in AD pathophysiology and the therapeutical role of MG remodeling [129]. Similarly, fecal microbiota transfer from wild type mice to AD mice alleviates AD hallmarks [130]. The modulation of the GM composition exerts a relevant role in modulating the activity of the microglia and its Aβ clearance capability [131]. Interestingly, the transplantation of GM from human AD patients to wild type mice results in gut inflammatory response [132]. AD patients show Aβ deposition also in the intestine [133].

Thus, GM participates in the regulation of the brain-heart axis by acting on several pathways modulating the ANS (Figure 1). GM and metabolic pathways also show a bi-directional mutual regulation, which in turn has an impact on the heart-brain axis and also contributes to AD.

## 9. Conclusions

**Figure 2:**
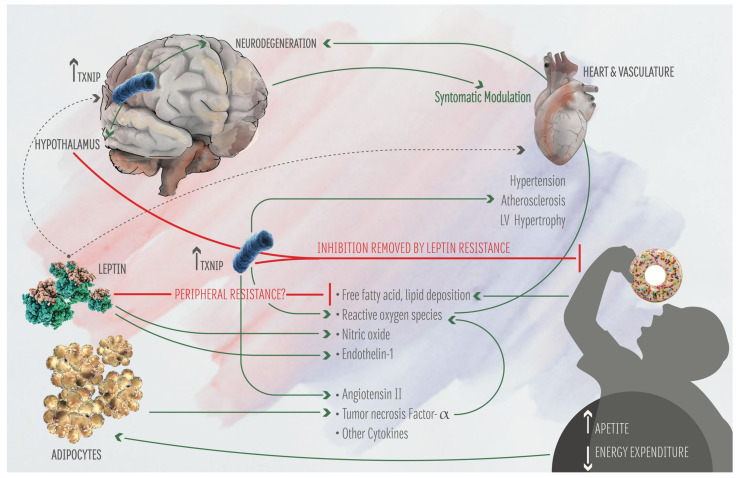
**Effect of TXNIP on the brain-heart axis and on human metabolism.** Food intake increases TXNIP expression in Agrp neurons of the hypothalamus, inducing satiety. Enhanced expression of TXNIP in the hippocampus leads to neuronal dysfunction. Hyperglycemia and diabetes induce elevated TXNIP expression in several tissues, leading to hypertension, atherosclerosis, and enhanced production of reactive oxygen species (ROS). In turn, ROS participate to neurodegeneration. It is also shown the effect of Leptin on the metabolism and the brain-heart axis.

The studies summarized above underline the bidirectional regulation between brain and heart as well as the key impact of metabolism in modulating the brain-heart axis. Notably, metabolic alterations have an effect on the brain-heart axis and participate to AD progression. Recent studies unveil the key role of metabolism in driving the early phases of AD. In addition, the study of the effect of metabolism in altering the brain-heart axis is providing new biomarkers for the early diagnosis of AD, such as ADMA and TXNIP. Moreover, recent evidences suggest that the analysis of TXNIP function may provide new therapeutic strategies for AD. The role of TXNIP in the progression of cardiovascular dysfunction as well as in the regulation of metabolism is well known, while the study of TXNIP implication in AD is more recent. Considering the role of TXNIP in mediating the whole-body response to metabolic alterations, the fact that TXNIP is a marker of cardiovascular diseases and is implicated also in AD, we may speculate that TXNIP has a key role in the brain-heart axis, and it may be implicated in driving the effect of metabolic alterations in brain-heart axis, which ultimately promote AD (Figure 2).

Further studies are important to consolidate the hypothesis that TXNIP drives the effect of metabolism in altering the brain-heart axis, leading a cascade of effects that in turn promote AD.

## Abbreviations

AD	Alzheimer Disease
TXNIP	Thioredoxin Interacting protein
Aβ	amyloid beta
APP	amyloid precursor protein
PSEN1 and PSEN2	presenilin 1 and 2
LOAD	late onset Alzheimer Disease
FAD	familiar Alzheimer Disease
NMDA	N-methyl-D-aspartate
ChEIs	inhibitors of the cholinesterase
ADMA	Asymmentric DiMethyl Arginine
SVD	small vessels disease
RAGE	Receptor for Advanced Glycation Endproducts
BBB	Blood Brain Barrier
Trx	Thioredoxin
ROS	reactive oxygen species
TBP-2	Thioredoxin Binding protein 2
VDUP-1	Vitamin D Upregulated Protein 1
AGEs	Advanced Glycation Endproducts
HMGB1	high mobiliy group B1
NLRP3	NOD-like receptor family, pyrin domain containing 3
PD	Parkinson Disease
HRV	heart rate variability
ApoE4	apolipoprotein E4
CAN	central autonomic network
ANS	autonomic nervous system
aMCI	amnestic mild cognitive impairment
HR	heart rate
ob	obese gene
db	diabetes gene
ObR	leptin receptor
BMI	body mass index
AGT	angiotensinogen
ANGII	angiotensin II
ACE1	angiotensin converting enzyme 1
ATRs	angiotensin receptors
PRR	pro-renin receptor
NPs	natriuretic peptides
ANP	atrial natriuretic peptide
BNP	brain natriuretic peptide
CNP	C-type natriuretic peptide
NPRA	Natriuretic Receptor A
NPRB	Natriuretic Receptor B
NPRC	Natriuretic Receptor C
NT-proBNP	N-terminal pro-brain natriuretic peptide
ET-1, 2, 3	Endothelin 1, 2, 3
PKC	Protein Kinase C
CNS	central nervous system
GM	gut microbiome
PAMPs	pathogen-associated molecular patterns
LPS	lipopolysaccharides
HFD	high-fat diet
MS	metabolic syndrome
GLP-1	glucagon-like peptide 1
GLP-1R	GLP-1 receptor
